# Effect of Nanofibrillated Cellulose Content on the Temperature and Near Infrared Responses of Polyvinyl Butyral Nanofibers-Containing Bilayer Hydrogel System

**DOI:** 10.3390/polym9070270

**Published:** 2017-07-06

**Authors:** Qian Zhao, Luping Ning, Yunhong Liang, Zhihui Zhang, Luquan Ren

**Affiliations:** 1 The Key Laboratory of Bionic Engineering, Ministry of Education, Jilin University, Changchun 130025, China; qianzhao16@mails.jlu.edu.cn (Q.Z.); ninglp16@mails.jlu.edu.cn (L.N.); zhzh@jlu.edu.cn (Z.Z.); lqren@jlu.edu.cn (L.R.); 2 State Key Laboratory of Automotive Simulation and Control, Jilin University, Changchun 130025, China

**Keywords:** bilayer hydrogel, multiple responses, electrospinning, nanofibrillated cellulose

## Abstract

A novel kind of nanofibrillated cellulose (NFC) reinforced polyvinyl butyral (PVB) nanofibers-containing bilayer hydrogel system was successfully fabricated via the combination of a one-step, in-situ, free radical polymerization and electrospinning. The hydrogel owned high mechanical strength, thermoresponsive, and near infrared bending/unbending properties. The cross-linking density of hydrogels enhanced along with the increase of NFC content. The addition of NFC and PVB nanofibers presented tiny influence on the variation of chemical bond and volume phase transition temperature. The combination between NFC and PVB nanofibers enhanced the mechanical strength and decreased the strain value, which built the base for high bonding strength of two layers and efficient thermoresponsive and near infrared responses. With the increase of NFC content, the bending degree became smaller. The bilayer hydrogel dimensions affected the deformation degree. Bilayer hydrogels with different NFC content own different deformation abilities, which can be designed as different parts of soft actuators and provide superior performance to satisfy various practical application demands.

## 1. Introduction

Attributed to the three-dimensional polymeric networks and remarkable capacity to absorb water, hydrogels have attracted significant attention and have been applied in medical science, technology, and soft material fields [1,2,3,4]. One of the most widely used hydrogels, thermoresponsive hydrogel fabricated from *N*-isopropylacrylamide (NIPAAm) and nanosized synthetic hectorite clay, are treated as material candidates for a soft robot, soft actuator, and 4D printing [5,6,7,8,9]. Based on a temperature-responsive hydrogel, the photoresponsive hydrogel is fabricated by the addition of gold nanorods, carbon nanotubes, graphene oxide [10,11,12], and so on to convert light into heat, which resulted in the size and shape change of hydrogel. Compared with temperature-responsive hydrogel, the photoresponsive one exhibits the characteristic of non-contact control, avoiding the restriction of environmental temperature. The photo-driving action can be actualized via the way of energy transformation based on light-absorbing nanoparticles embedded in a thermosensitive hydrogel. In virtue of practicality, convenience, and better-suited to biological applications, [13] the near-infrared (NIR) light is treated as the manipulator of photoresponsive hydrogel. Recently, due to the high photothermal energy transformation and absorbance of NIR energy, [14,15] graphene oxide (GO) has been wildly utilized in photoresponse hydrogels.

Mechanical strength is a basic characteristic for the application of hydrogels. In order to improve the mechanical properties, as a kind of filler, nanofibrillated cellulose (NFC) has been added into the hydrogel matrix to provide a kind of hydrogel material with high strength for biomimetic 4D printing [9]. As a kind of method for increasing mechanical strength, nanofiber reinforced composite material provided another way to increase the mechanical strength of hydrogels. With the development of a nanofiber fabrication method, electrospinning is regarded as a simple and versatile method for generating nanofibers with a unique advantage of high surface area, which has been treated as a best candidate in many different practical applications [16,17,18]. As a result of a relatively high mechanical strength and anti-impact properties, polyvinyl butyral is treated as a kind of electrospinning material [19]. Therefore, in the view of material composition and material structure, we try to add the nanofibrillated cellulose and the nanofibers obtained from electrospinning into the hydrogel with temperature and near infrared responses to improve the corresponding mechanical strength.

Structure modality is the key point for a hydrogel material to achieve bending/unbending ability. Wang and co-workers [20] synthesized near-infrared light-driven hydrogels actuators by interfacing genetically engineered elastin-like polypeptides with reduced-graphene oxide sheets, which exhibited rapid and tunable motions controlled by light position, intensity, and path, including finger-like flexing and crawling. The common ground of the responsive bending of hydrogels are the anisotropic responses of layered structure. The model of two layers with different temperature or near infrared absorbing capacities is the simplest layered structure. In order to achieve the smart bending properties, some kind of bilayer structure consisting of a temperature and photoresponse hydrogel layer and a nonresponsive layer are fabricated via in-situ, free radical polymerization. Jiang and co-workers [21] designed and fabricated a photoresponsive biomimetic microfish, which can move forward, backward, and turn around in water under near infrared irradiation. Yao and co-workers [5] fabricated a double-layered temperature-controlled hydrogel with relative high responsive bending and elastic properties via a two-step photopolymerization. This kind of hydrogel can be treated as a temperature-controlled manipulator for various applications including encapsulation, capture, and transportation of targeted objects. Even though the temperature and photoresponse behaviors of hydrogels with layered structures were investigated [5,6], how to combine electrospinning and realizing the simple fabrication method of one-step, in-situ, free radical polymerization has not been investigated.

In this paper, by regulating the nanofibrillated cellulose content, we fabricated a series of polyvinyl butyral-containing bilayer hydrogel system with temperature and near infrared responses via a one-step, in-situ, free radical polymerization and electrospinning. Variation of nanofibrillated cellulose content affected cross-link density of hydrogels, which built the fabrication base for the one-step, in-situ, free radical polymerization. The incorporation of nanofibrillated cellulose and polyvinyl butyral nanofibers enhanced mechanical strength and maintained the bending/unbending properties under the stimulation of temperature and a near infrared laser. The bilayer hydrogel with different strength and bending degree can be treated as a kind of smart material for soft actuators and robots driven by temperature and near infrared laser.

## 2. Experimental Section

### 2.1. Material

Monomer *N*-isopropylacrylamide (NIPAAm, C_6_H_11_NO, Aladdin, Shanghai, China, 2% stabilizer) was recrystallized from toluene/*n*-hexane mixture and dried in vacuum at room temperature for 48 h. Nanosized synthetic hectorite clay, Laponite XLG, Mg_5.34_Li_0.66_Si_8_O_20_(OH)_4_ was purchased from Rockwood, Ltd., (Moosburg, Germany) and used after drying at 125 °C for 2 h. Initiator potassium peroxydisulfate (KPS, K_2_S_2_O_8_, Shanghai Aibi Chemical Reagent Co., Ltd., Shanghai, China, Analytical reagent AR), catalyst *N*,*N*,*N*′,*N*′-tetramethylethylenediamine (TEMED, Tianjin Weiyi Chemical Technology Co., Ltd., Tianjin, China, 98%), graphene oxide (GO, Suzhou Hengqiu Graphene Technology Co., Ltd., Suzhou, China, 95%), Nanofibrillated cellulose (NFC, Guilin Qihong Technology Co., Ltd., Guilin, China, 1342 nm) and methyl blue (Analytical reagent AR, Shanghai Aibi Chemical Reagent Co., Ltd., Shanghai, China) were used as received. The polyvinyl butyral (PVB, aircraft-grade) and ethyl alcohol (Analytical reagent AR) used for electrospinning were purchased from Sinopharm Chemical Reagent Co., Ltd. (Shanghai, China). Pure water was obtained by deionization and filtration with a Millipore purification apparatus (resistivity ≥ 18.2 MΩ·cm). 

### 2.2. Synthesis of PNIPAm–NFC Hydrogel

**Figure 1:**
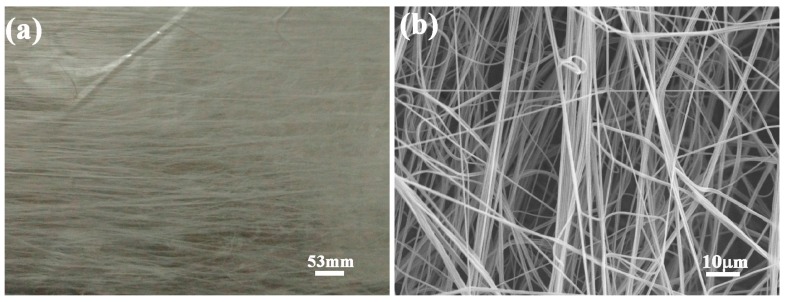
(**a**) Morphology and (**b**) SEM microstructure of PVB nanofibers fabricated via electrospun.

Before the synthesis of hydrogels, PVB nanofibers were fabricated via electrospinning (Model SS-2535H, Beijing Uclalery Technology Development Co., Ltd., Beijing, China). The homogeneous and stable PVB solution with concentration of 7% and weight of 20 g was prepared at room temperature. In order to ensure the oriented characteristics of PVB nanofibers, model of conjugate spinning was employed. The positive high voltage of 10.8 kV and negative high voltage of −10.8 kV were applied to the right and left syringe needles of the electrospinning machine, respectively. The distance between the tip of the needle and the grounded collector was fixed at 10 cm. Figure 1 shows a typical scanning electron microscopy (SEM) image of electrospun PVB nanofibers. The nanofibers exhibited the oriented characteristics on the substrate, forming an interwoven network structure. 

**Figure 2:**
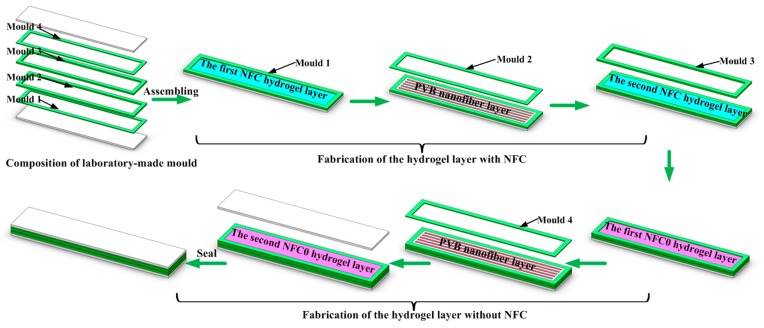
The diagrammatic sketch of the fabrication process of PVB-containing bilayer hydrogel systems with various NFC contents.

The PNIPAm–NFC hydrogel was synthesized via in-situ, free radical polymerization of NIPAm in the nanosized clay suspension with GO and NFC. Before the fabrication of PNIPAm–NFC hydrogel, the pure water was degassed in the continuous nitrogen-saturated atmosphere for 2 h. Then 0.5 mL of methyl blue solution with a concentration of 40 mg/mL was stirred for 0.5 h in 19 mL pure water for the fabrication of the hydrogel without GO and NFC. The graphene oxide was first dispersed in 19.5 mL of pure water by ultrasonic radiation for 30 min and stirred for 1 h via a magnetic stirrer (Model DF-101S, Changchun Jiyu Technology Equipment Co., Ltd., Changchun, China). The XLG clay was added into GO suspension, which was stirred for 1 h and ultrasonically radiated for 30 min. Then NFC, with a corresponding weight, was added and stirred for 1 h in an ice-water bath. The monomer NIPAm was added into miscible liquids of GO, XLG, and NFC under a nitrogen-saturated atmosphere in an ice-water bath for another 2 h. Finally, 0.5 mL of KPS solution with a concentration of 40 mg/mL and 27 μL of the catalyst TEMED was added under stirring. The solution was rapidly injected into a laboratory-made rubber mold of 70 mm × 20 mm × 2 mm (Length × Width × Thickness). The polymerization was conducted at 25 °C for 24 h to produce the NC hydrogel. The mole ratio of NIPAm monomer, initiator, and catalyst in all suspensions was kept at 100:0.370:0.638. Under the photothermal energy transformation property of GO, in order to investigate the effect of NFC content on the microstructure, mechanical properties and temperature, and near infrared response of the hydrogel, 0, 1, 2 and 3 mg/mL NFC was added in the hydrogel, respectively. In this paper, the PNIPAm–NFC hydrogels were defined as NFC0, NFC1–PVB, NFC2–PVB, and NFC3–PVB, where 0, 1, 2, and 3 represented the concentration of NFC. The compositions of the hydrogels are listed in Table 1. In order to investigate the temperature and near infrared responses of the bilayer hydrogel system, the corresponding layered structure was fabricated, as shown in Figure 2. In the bilayer structure, due to the existence of GO, the layer with NFC consisted of two NFC hydrogel layers and a PVB nanofiber layer, which presented black. Attributed to the existence of methyl blue, the layer without NFC consisted of two NFC0 hydrogel layers and a PVB nanofiber layer, which exhibited blue. The NFC contents were 1, 2 and 3 mg/mL, respectively. Due to the variation of NFC, the two layers of the bilayer hydrogel system exhibited different densities, which provided a method for fabrication of the bilayer hydrogel system efficiency via density difference in a one-step, in-situ, free radical polymerization. The layer with NFC was injected first in the laboratory-made rubber mold. The PVB nanofiber layer was paved in the middle of the NFC hydrogel layer. After the superimposition of NFC0 hydrogel layers and another PVB nanofiber layer, the laboratory-made mold was sealed tightly. The polymerization of the bilayer hydrogel system was also carried out at 25 °C for 24 h. 

### 2.3. Characteristics

#### 2.3.1. Microstructure

In order to observe the internal microstructure via a scanning electron microscope (SEM) (Model Evo18 Carl Zeiss, Oberkochen, Germany) and an environmental scanning electron microscope (ESEM-FEG) (Model XL-30, FEI Company, Hillsboro, OR, USA), the corresponding samples with dimensions of 5 mm × 5 mm × 2 mm (Length × Width × Thickness) were placed in a freeze drying oven (LGJ-10C, Beijing Four Ring Scientific Instrument Factory Co., Ltd., Beijing, China) to remove water thoroughly. After gold sputtering, the cross section of samples was observed with magnifications of 200×, 5000×, and 20,000×, respectively. 

#### 2.3.2. Infrared Spectrum and Differential Scanning Calorimetry (DSC) Analysis

Fourier transform infrared (FT-IR) spectra of hydrogels were analyzed via IRAffinity-1 FT-IR spectrometer (Shimadzu Corporation, Kyoto, Japan). The range of wavenumber was 500–4000 cm^−1^. Before measurement, the samples were ground into powder and pressed with KBr into a disc.

The volume phase transition temperature (VPTT) of the hydrogel sample was measured via differential scanning calorimetry (DSC) (Model Q2000, TA Company, Boston, PA, USA). The samples were heated from 20 to 50 °C, and then cooled from 50 to 20 °C at the rate of 10 °C/min under the nitrogen atmosphere. The VPTT of the hydrogel was determined at the onset of the endotherm peak during second heating.

#### 2.3.3. Tensile Stress-Strain Analysis

To obtain the tensile stress-strain characteristic of hydrogels, a universal testing machine (Model C43, MTS Criterion, Eden Prairie, MN, USA) with the constant loading rate of 100 mm/min was employed to test the tensile properties. The sample size was 60 mm × 6 mm × 2 mm (Length × Width × Thickness). Average values of stress and strain were calculated from three individual measurements.

#### 2.3.4. Temperature and Near Infrared Responses

In order to disclose the effect of NFC content and sample size on dynamic thermoresponsive bending behaviors, the completely swollen bilayer hydrogels with different NFC contents were cut into strips of 40 mm × 8 mm × 4 mm and 70 mm × 5 mm × 4 mm (Length × Width × Thickness), respectively. The bilayer hydrogels were placed in water of poikilothermy temperature ranging from 30 to 50 °C. The whole dynamic thermoresponsive bending process was recorded by a digital camera. The near infrared response of the hydrogel system was conducted via a near infrared illuminant with a wavelength of 808 nm and power of 3 W (Model FC-W-808-30W, Changchun New Industries Optoelectronics Technology Co., Ltd., Changchun, China). The distance between the light source and sample was 50 cm. The energy density delivered to the sample was 3.82 W/cm^2^. The deformation and recovery process of the hydrogel system were conducted in pure water at 25 °C. A digital camera was used to record the whole dynamic infrared driving bending process.

## 3. Results and Discussion

### 3.1. Microstructure of PNIPAm–GO Hydrogel

**Figure 3:**
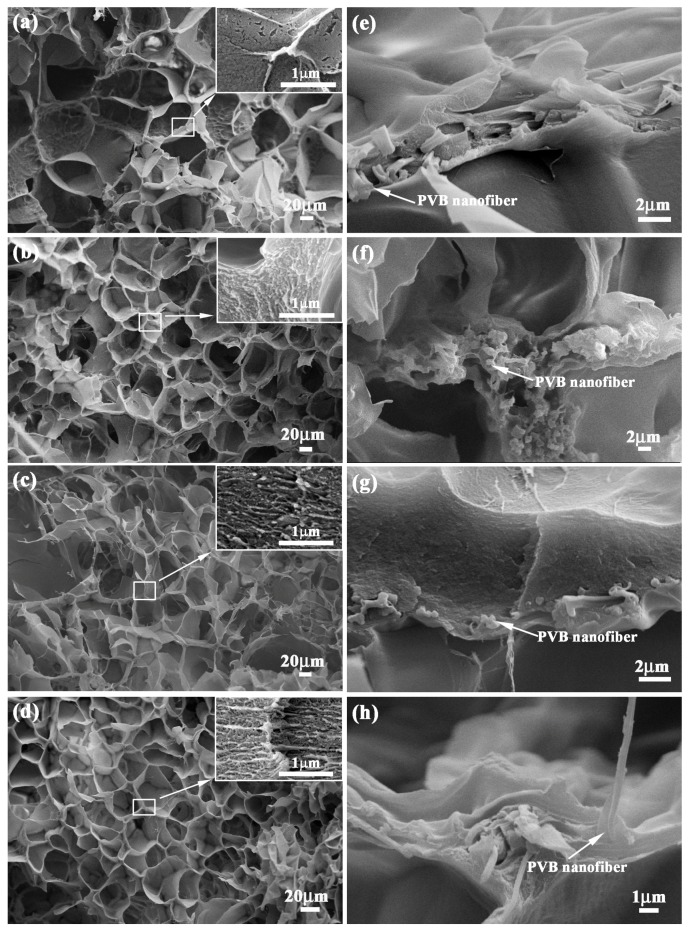
Microstructure of PVB-containing hydrogels with (**a**) 0 mg/mL, (**b**) 1 mg/mL, (**c**) 2 mg/mL and (**d**) 3 mg/mL NFC and the distribution pattern of PVB nanofibers in corresponding hydrogels with (**e**) 0 mg/mL, (**f**) 1 mg/mL, (**g**) 2 mg/mL, and (**h**) 3 mg/mL NFC.

Figure 3a–d show the microstructure of PVB-containing hydrogels with 0, 1, 2 and 3 mg/mL NFC contents. It can be observed that the PNIPAm hydrogels presented a honeycomb-like structure and uniform distribution. The pore size became smaller with the increase of NFC contents qualitatively, indicating the enhancement of cross-linking density in the hydrogel network. Moreover, the size of net structure in the high magnification became smaller along with the increase of NFC contents. The reinforcement of the PVB nanofibers exhibited an excellent combination with the hydrogel, as shown in Figure 3e–h. PVB nanofibers maintained the morphology which was similar to Figure 1b. The addition of NFC and PVB nanofibers exhibited a tiny influence on the synthesis of hydrogels.

### 3.2. FT-IR Spectra and DSC Analysis

**Figure 4:**
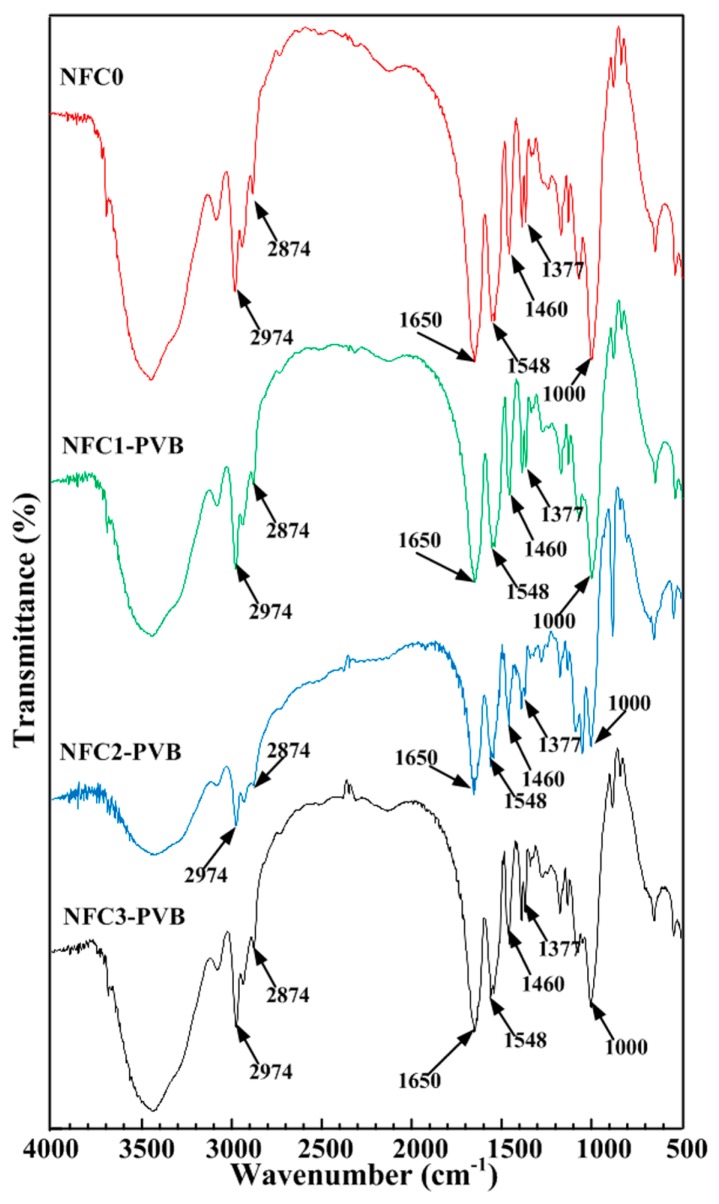
Typical FT-IR spectra characteristics of PVB-containing hydrogels with various NFC contents.

In order to disclose whether the addition of NFC affected the existence of acylamino and isopropyl in hydrogels or not, a Fourier transform infrared spectra was conducted, In Figure 4, the 2974, 2874, 1460, and 1377 cm^−1^ were the peaks of –CH_3_. The peak of 1377 cm^−1^ was divided into two peaks, which exhibited the characteristic of isopropyl. The C=O stretching vibration peak of 1650 cm^−1^ and N–H bending vibration peak of 1548 cm^−1^ were the characteristic peaks of amide. The stretching vibration peak of C–O–C in 1000 cm^−1^ expressed the existence of GO in hydrogels. Variation of NFC content exhibited a relatively tiny influence on the FT-IR profiles. Figure 4 disclosed the appearance of hydrophilic acylamino and the hydrophobic isopropyl of *N*-isopropylacrylamide, which built the functional base for the thermoresponsive and near infrared laser characteristic. 

**Figure 5:**
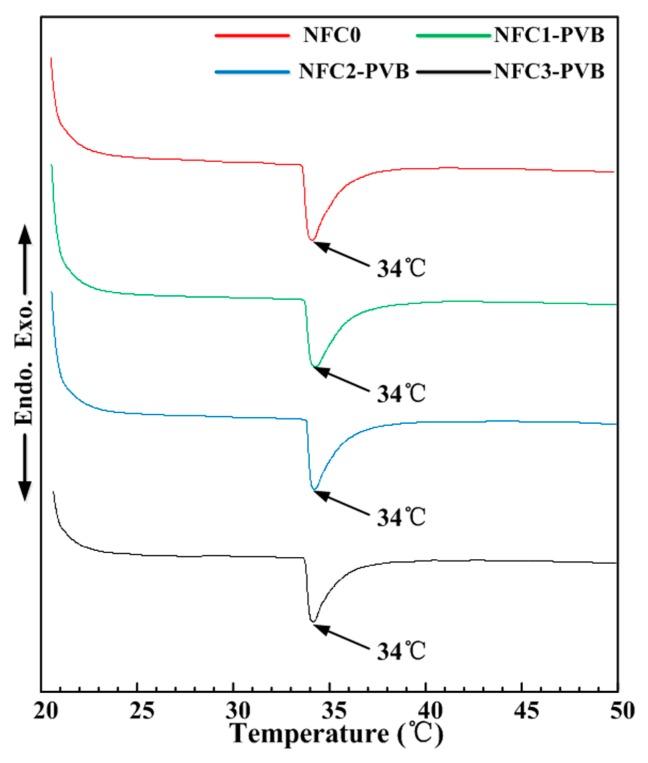
DSC profiles of PVB-containing hydrogels with various NFC contents.

Figure 5 shows the DSC profiles of hydrogels with various NFC contents. The VPTT is a constant at about 34 °C. When the temperature was higher than 34 °C, the hydrogels presented volume shrink. Compared with the NFC0 hydrogel, no obvious variation in VPTT can be found in that of NFC–PVB hydrogels. Therefore, the addition of PVB nanofibers and variation NFC presented a tiny influence on the change of volume phase transition temperature of hydrogels. The hydrogels possessed the well-defined phase transition temperature, which provided the thermal sensitivity for potential applications as an actuation material. The PNIPAm–NFC hydrogels showed a typical honeycomb-like structure, complete chemical bonds, and a steady VPTT, which owned the material base for thermoresponsive and near infrared response. Besides chemical bonds, mechanical strength was another important factor for applications. Therefore, mechanical experiments were conducted to find the effect of NFC content on the mechanical strength.

### 3.3. Mechanical Properties

Average values of stress, strain and modulus of PVB nanofibers-containing hydrogels with various NFC contents are shown in Table 2. The addition of NFC and PVB nanofibers enhanced the mechanical strength and decreased strain values. The variation of NFC content excellently affected the corresponding mechanical strength. With the increase of NFC content, the stress and strain values increased and decreased, respectively. The stress values of NFC0, NFC1–PVB, NFC2–PVB, and NFC3–PVB hydrogels are 12.9, 22.1, 23.1, and 24.0 KPa, respectively. The corresponding strain values are 994.7%, 735.5%, 688.6%, and 502.3%, respectively. The hydrogel with 3 mg/mL NFC content presented the highest strength and lowest strain. 

Combined with Figure 3, it can be found that the variation of NFC content enhanced the cross-link density of hydrogels. Moreover, the distribution direction of PVB nanofibers exhibited high bonding strength with hydrogel base along with tension force direction. The combinations between NFC and PVB nanofibers enhanced the mechanical strength. The different microstructure and mechanical strength of the hydrogels with various NFC content provided the feasibility for the design and fabrication of a bilayer hydrogel system. In order to study the temperature and near infrared responses characteristics of bilayer hydrogel systems, the corresponding fabrication and analysis were conducted.

### 3.4. Microstructure and Responsive Characteristics of Bilayer Hydrogel System

#### 3.4.1. Microstructure Characteristic

**Figure 6:**
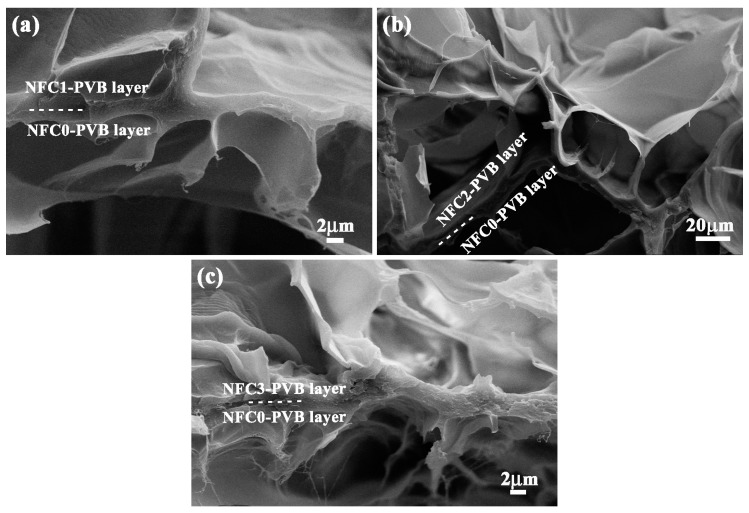
Interface bonding characteristic of (**a**) NFC1–PVB, (**b**) NFC2–PVB, and (**c**) NFC3–PVB bilayer hydrogels.

In order to disclose the bonding condition between the NFC–PVB layer and the NFC0 layer, the bonding interface of the bilayer hydrogel systems was observed, as shown in Figure 6a–c. Due to the difference of the honeycomb-like structure size between the two layers, the boundary can be observed and marked in the form of an imaginary line, which exhibited high bonding strength of the bilayer hydrogel system. The NFC1, NFC2, and NFC3 bilayer hydrogel systems exhibited a uniform porous network, which was similar to the microstructure in Figure 3. Compared with the NFC0 layer, the NFC-containing hydrogel layers exhibited a relatively compact morphology. With the increase of the NFC content, the size of the honeycomb-like structure decreased gradually, which was identical to that of the unilaminar hydrogels. The change tendency of structure characteristics of the bilayer hydrogel systems with different NFC content was irrelevant to the layered combination method. As a result of plenty of crosslinks across the interface, the two layers were locked together tightly. The steady bonding strength built a substantial base for the application of the bilayer hydrogel systems with high mechanical strength driven by temperature and near infrared laser stimulation. Besides the thermoresponsive and near infrared response, the effects of sample size on deformations were also conducted. 

#### 3.4.2. Temperature Responsive Properties

**Figure 7:**
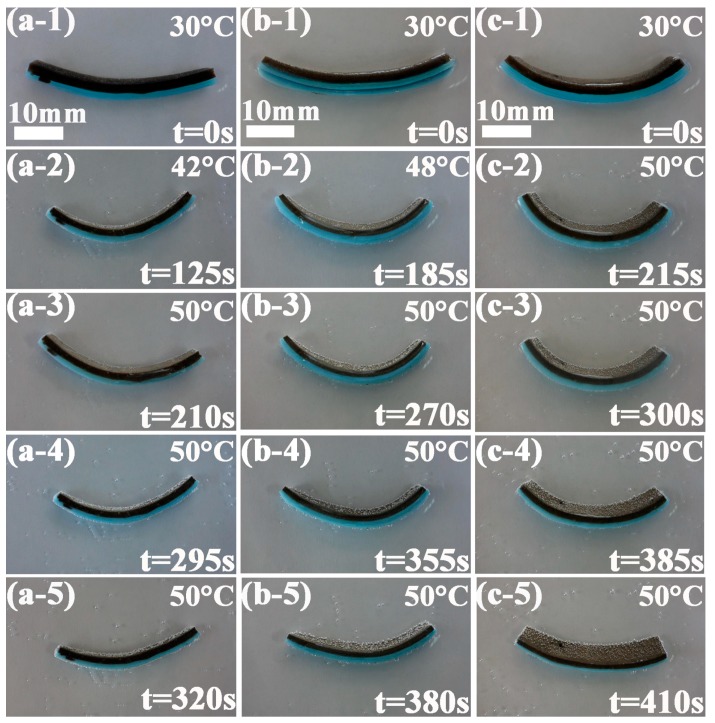
Thermoresponsive characteristics of (**a**-**1**)–(**a**-**5**) NFC1, (**b**-**1**)–(**b**-**5**) NFC2, and (**c**-**1**)–(**c**-**5**) NFC3 of PVB nanofibers-containing bilayer hydrogel systems with dimensions of 40 mm × 8 mm × 4 mm (Length × Width × Thickness) at poikilothermy temperatures ranging from 32 to 50 °C.

The dynamic thermoresponsive bending behaviors of PVB nanofiber-containing bilayer hydrogel systems with dimensions of 40 mm × 8 mm × 4 mm (Length × Width × Thickness) at a poikilothermy temperature ranging from 32 to 50 °C are shown in Figure 7 (a-1)–(c-5). The heating rate was 6 °C/min. The time marked in Figure 7 represents the time of the whole bending and unbending process. The corresponding first figure in (a-1)–(a-5), (b-1)–(b-5) and (c-1)–(c-5) is the initial state. The second and third figures are the bending process. The fourth and fifth figures reflect the expansion process. The NFC1–PVB bilayer hydrogel strip bent toward the NFC1 layer in Figure 7 (a-1)–(a-5). With time going on, the NFC1–PVB bilayer hydrogel strip gradually reached the maximum bending degree at the beginning of 210 s in Figure 7 (a-3). Then the bilayer hydrogel system changed from bending state to unbending state, and was in the progress of a gradual expansion. The NFC2–PVB nanofibers and the NFC3–PVB nanofibers bilayer hydrogel strips exhibited the similar bending/unbending phenomenon, but the corresponding distinctive characteristics presented the influence of the NFC content. With the increase of the NFC content, the bending degree became smaller qualitatively, and the time to reach maximum bending degree became longer. The NFC3–PVB bilayer hydrogel owned the relatively lowest bending property. 

The combination among the microstructure and mechanical properties results of the NFC–PVB bilayer hydrogels significantly affected the thermoresponsive bending property. The cross-link density of the hydrogels increased along with the increase of the NFC content, leading to the decrease of the honeycomb-like structure size and movement capacity of the polymer chain. Therefore, the bending ability of the bilayer hydrogels decreased with the increase of the NFC content.

**Figure 8:**
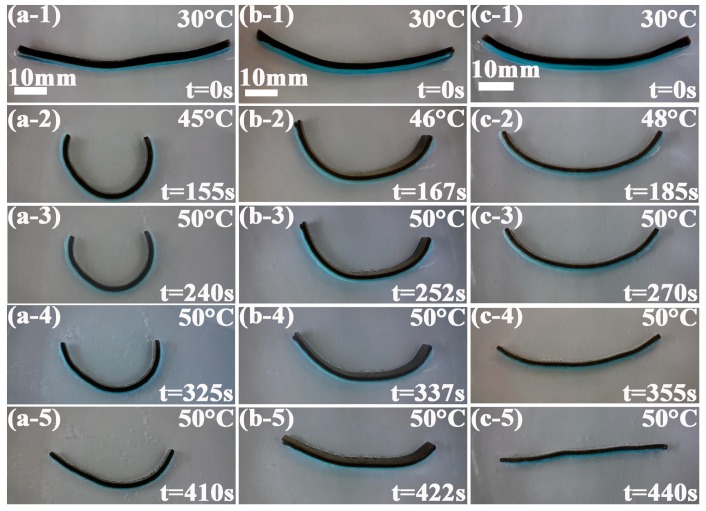
Thermoresponsive characteristics of (**a**-**1**)–(**a**-**5**) NFC1–PVB, (**b**-**1**)–(**b**-**5**) NFC2–PVB, and (**c**-**1**)–(**c**-**5**) NFC3–PVB bilayer hydrogel systems with dimensions of 70 mm × 5 mm × 4 mm (Length × Width × Thickness) at poikilothermy temperatures ranging from 30 to 50 °C.

After the investigation of thermoresponsive characteristics of the bilayer hydrogel systems along with the variation of the NFC content, the influence of sample size on the bending degree was analyzed. Thermoresponsive characteristics of the NFC1–PVB, NFC2–PVB, and NFC3–PVB bilayer hydrogel systems with dimensions of 70 mm × 5 mm × 4 mm (Length × Width × Thickness) at poikilothermy temperatures ranging from 30 to 50 °C are shown in Figure 8 (a-1)–(a-5), (b-1)–(b-5), and (c-1)–(c-5). From Figure 8 (a-1)–(a-5), it can be found that with the progress of the experiment, the bilayer hydrogel bent toward the NFC layer, and changed from straight state to semicircle, which can be attributed to the different cross-link density properties of the NFC1 and NFC0 hydrogel layers. The NFC1–PVB bilayer hydrogel system exhibited the highest bending degree. The time NFC1 bilayer hydrogel used to get a maximum bending degree at 240 s. After 85 and 170 s. respectively, the bilayer hydrogel changed from semicircle state to straight state, and was in the progress of expansion gradually. The NFC2–PVB and NFC3–PVB bilayer hydrogel used 252 and 270 s to bend from the initial state to the maximum bending degree, as shown in Figure 8 (b-3) and (c-3), which was longer than that of the NFC1–PVB one. Compared with Figure 7, when the sample dimensions changed from 40 mm × 8 mm × 4 mm to 70 mm × 5 mm × 4 mm, the bilayer hydrogels exhibited different bending characteristics. The NFC-PVB bilayer hydrogel with a longer dimension in length and a smaller dimension in width owned a higher bending degree. In the expansion process, the NFC3–PVB bilayer hydrogel showed relative complete unbending behavior and obtained a straight state.

The dimensions of the bilayer hydrogel affected the thermoresponsive bending degree. The longer and narrower the dimensions of the bilayer hydrogel, the higher the bending degree of the bilayer hydrogel exhibited, but the effect of the NFC content on bending degree remained unchanged. The role of the dimensions of the bilayer hydrogel can be provided for the design direction of soft actuator fabrication.

#### 3.4.3. Near Infrared Responsive Characteristic

**Figure 9:**
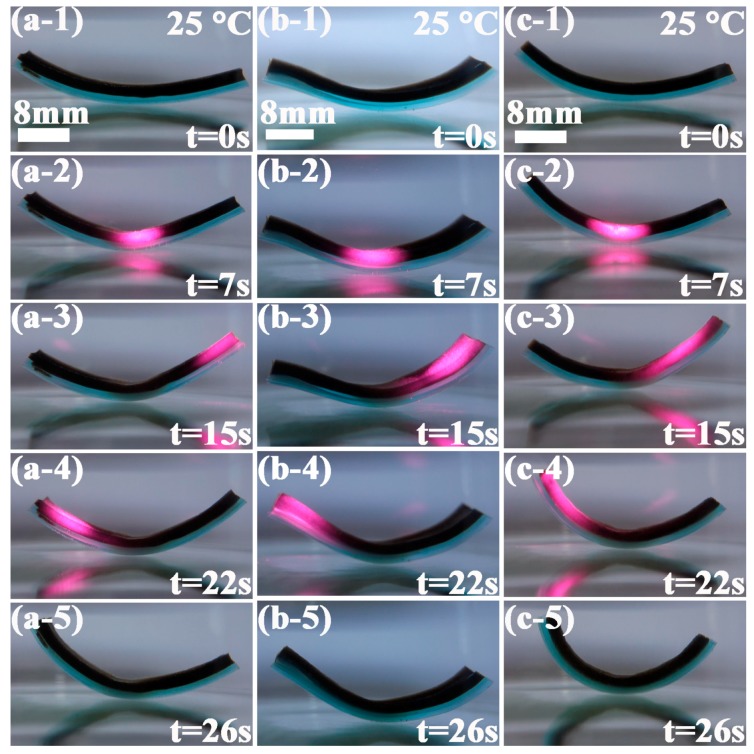
Near infrared responsive characteristic of bilayer hydrogel systems with different NFC contents (**a-1**)–(**a-5**) NFC1–PVB, (**b-1**)–(**b-5**) NFC2–PVB, and (**c-1**)–(**c-5**) NFC3–PVB in a different period.

In order to investigate the effect of NFC content on near infrared responsive characteristic, bilayer hydrogels with dimensions of 40 mm × 8 mm × 4 mm (Length × Width × Thickness) were irradiated by a near infrared laser. Figure 9 shows the dynamic near infrared bending and shrinking characteristics in water with a temperature of 25 °C. Grapheme oxide in the hydrogel absorbed the near infrared laser and transformed the photoenergy to thermal energy rapidly and efficiently, resulting in the faster temperature increase in the NFC hydrogel layer than that of NFC0 layer. Therefore, the bilayer hydrogel bent toward the NFC layer side. The time marked in Figure 9 represented the time for irradiation of the near infrared laser in different parts of the bilayer hydrogels. As shown in Figure 9 (a1)–(a5), under the raying of near infrared on the middle part, the right and left part of the NFC1–PVB bilayer hydrogel bent to the middle part. When the near infrared moved to the right part, the corresponding part bent to the left direction. Under the raying of near infrared, the left part bent to the right direction. After removing near infrared, the unbending process was conducted and became straight gradually. From Figure 9 (b-1)–(b-5) and (c-1)–(c-5), it can be found that the NFC2–PVB and NFC3–PVB bilayer hydrogel presented the similar bending property and corresponding individual peculiarities. With the increase of NFC content, the bending degree became lower qualitatively. The NFC3–PVB bilayer hydrogel exhibited the lowest bending property. The reasons for the bending phenomenon of the bilayer hydrogels under initiation of near infrared were similar to those of the thermoresponsive bending experiment. The increase of the NFC content enhanced the cross-link density and decreased the network size, movement capacity, and bending ability of the polymer chain in the bilayer hydrogel. 

Combined with the characteristics of microstructure, mechanical strength, and bending/unbending, the bilayer hydrogel system exhibited high thermoresponsive and near infrared response, which proved the feasibility of the design and fabrication of a layered structure constituted with various NFC content. The bilayer hydrogel systems with various mechanical strength own different deformation abilities, which can be designed as different parts of soft actuators and provide wide material choices for soft-robotics. 

## 4. Conclusions

By regulating the NFC content, a novel kind of PVB nanofiber-containing bilayer hydrogel system with excellent temperature and near infrared responses was successively fabricated via the combination of a one-step, in-situ, free radical polymerization and electrospinning. The PVB nanofibers showed high combination with the hydrogel base with a typical honeycomb-like structure. The size of the microstructure became smaller with the increase of the NFC contents, indicating the increase in cross-linking density. The addition and variation of the NFC and PVB nanofibers presented inessential influence on the change of the chemical bond and volume phase transition temperature. The combination between the NFC and PVB nanofibers enhanced the mechanical strength and decreased the strain value, which built the base for high bonding strength between the two layers and an efficient thermoresponsive and near infrared responsive bending/unbending. With the increase of the NFC content, the bending degree became smaller. Sample dimensions affected the deformation degree. The longer the dimension in length and smaller the dimension in width, the higher bending degree the bilayer hydrogel exhibited. Different NFC-containing bilayer hydrogels own different deformation abilities, which can be designed as different parts of soft actuators and provide superior performance to satisfy various practical application demands.

## Figures and Tables

**Table 1:** Compositions of PNIPAm–NFC hydrogels.

Sample	NIPAm (g)	GO (mg)	NFC (mg)	XLG (g)	KPS (mg)	TEMED (μL)	H_2_O (mL)
NFC0	2.26	20	0	0.693	20	27	20
NFC1–PVB	2.26	20	20	0.693	20	27	20
NFC2–PVB	2.26	20	40	0.693	20	27	20
NFC3–PVB	2.26	20	60	0.693	20	27	20

**Table 2:** Average values of stress, strain, and modulus of PVB nanofibers-containing hydrogels with various NFC contents.

Sample	Stress (KPa)	Strain (%)	Modulus (Pa)
NFC0	12.9 ± 3.6	994.8 ±87.1	13.2 ± 4.8
NFC1–PVB	22.1 ± 2.1	735.6 ±40.7	30.2 ± 4.5
NFC2–PVB	23.1 ± 3.5	688.6 ± 45.6	33.9 ± 7.3
NFC3–PVB	24.0 ± 2.5	502.3 ± 45.3	48.3 ± 9.4
